# Effects of the multidomain non-pharmacological interventions on anxiety symptoms and depressive symptoms in older adults with suspected cognitive impairment: a propensity score matching and difference-in-differences analysis

**DOI:** 10.3389/fnagi.2026.1921356

**Published:** 2026-08-21

**Authors:** Yu Zhang, Tong Li, Ziyi Chen, Peng Zeng, Yu Su, Yong Zhao

**Affiliations:** 1 Office of Party Affairs, Chongqing Jiulongpo District Center for Disease Control and Prevention, Chongqing, China; 2 School of Public Health, Chongqing Medical University, Chongqing, China; 3 Research Center for Medicine and Social Development, Chongqing Medical University, Chongqing, China; 4 Research Center for Public Health Security, Chongqing Medical University, Chongqing, China; 5 Nutrition Innovation Platform-Sichuan and Chongqing, School of Public Health, Chongqing Medical University, Chongqing, China; 6 College of Traditional Chinese Medicine, Chongqing Medical University, Chongqing, China; 7 Department of Health Education and Health Promotion, Chongqing Jiulongpo District Center for Disease Control and Prevention, Chongqing, China; 8 Chongqing Key Laboratory of Child Nutrition and Health, Children’s Hospital of Chongqing Medical University, Chongqing, China

**Keywords:** anxiety symptoms, depressive symptoms, intervention study, suspected cognitive impairment, the multidomain non-pharmacological interventions

## Abstract

**Background:**

Mental health problems among older adults with cognitive impairment have attracted increasing attention. Although non-pharmacological interventions (NPIs) are considered safe and feasible, evidence regarding their effects on older adults with suspected cognitive impairment in Southwest China remains limited.

**Methods:**

This intervention study was conducted in an urban district of Chongqing, China, including older adults with suspected cognitive impairment. A total of 159 individuals were assigned to the intervention group and 161 to the control group. The intervention group received routine health education in addition to a 2-month multidomain NPIs, while the control group received routine health education only. The multidomain NPIs included multiple components, such as music, physical exercise, and sensory stimulation. Data were collected through face-to-face interviews using validated assessment tools, including the Generalized Anxiety Disorder Scale and the 15-item Geriatric Depression Scale. Propensity score matching (PSM) was used to balance baseline characteristics, and difference-in-differences (DID) models estimated the net intervention effect. Difference-in-Difference-in-Differences (DDD) models further examined whether baseline cognitive status modified the effects on anxiety and depressive symptoms.

**Results:**

In this study, the prevalence of anxiety and depressive symptoms was 24.38% and 10.94%, respectively. After PSM, 202 participants were included in the analysis. DID results showed that the multidomain NPIs significantly reduced anxiety symptoms (β = −1.44, 95% CI: −2.22, −0.65, *P* < 0.001) and depressive symptoms (β = −2.04, 95% CI: −2.83, −1.25, *P* < 0.001) among older adults with suspected cognitive impairment. Model 3 of the DDD analysis indicated that baseline cognitive status significantly moderated the effect of the multidomain NPIs on depressive symptoms (Treatment × Time × Cognitive status: β = 2.14, 95% CI: 0.25–4.04, *P* = 0.027). These findings suggest that, compared with older adults with baseline cognitive impairment, the intervention showed a greater effect on reducing depressive symptoms among older adults without objective cognitive impairment at baseline.

**Conclusion:**

The multidomain NPIs may reduce anxiety and depressive symptoms in older adults with suspected cognitive impairment, with effects on depressive symptoms potentially moderated by baseline cognitive status. Integrating the multidomain NPIs into early cognitive impairment management may improve emotional well-being.

## 1 Introduction

The global population is aging rapidly, and the prevalence of age-related cognitive impairment is rising. Among people aged 65 and older, 30.0%–40.0% are estimated to experience cognitive decline (Alzheimer’s Association and CDC, 2025). Cognitive impairment is an age-associated neurodegenerative condition characterized by progressive declines in memory, learning capacity, and attention, and it may eventually progress to dementia (World Health Organization [WHO], 2010). Dementia ranks as the seventh leading cause of death worldwide and is a major contributor to loss of independence and daily functioning in older adults (World Health Organization [WHO], 2025a). The global prevalence of cognitive impairment ranges from 5.1% to 41.0%, with Europe reporting 5.1%–41.0% and Asia 6.5%–37.0% (Pais et al., 2020). A 2024 survey in China found that 28.4% of older adults had mild cognitive impairment (Liu et al., 2025). Cognitive impairment can seriously affect health and quality of life and may increase the risk of death. The Lancet Commission on dementia prevention, intervention, and care reported that addressing 14 modifiable risk factors may prevent or delay nearly half of dementia cases, including hypertension, obesity, diabetes, hearing loss, and depression (Livingston et al., 2024). Mental health in people with cognitive impairment has drawn growing research interest. Systematic reviews and meta-analyses have reported prevalence rates of 21.0% for anxiety symptoms and 32.0% for depressive symptoms among patients with mild cognitive impairment (Chen et al., 2018; Ismail et al., 2017).

Depression is one of the most common mental disorders worldwide. It is marked by persistent low mood, loss of interest, reduced energy, poor concentration, and thoughts of death or suicide (American Psychiatric Association [APA] and DTF, 2013). Anxiety is a future-oriented emotional state. It involves anticipatory cognitive, behavioral, and emotional responses to uncertainty about potential threats (Grupe and Nitschke, 2013). More than 20.0% of adults aged 60 years and older worldwide are affected by neuropsychiatric conditions, including dementia, depressive symptoms, and anxiety symptoms (World Health Organization [WHO], 2025b). In China, depressive and anxiety symptoms are common mental health problems among older adults, with reported prevalence rates of 13.8% and 8.0%, respectively (Gao et al., 2025). Anxiety and depressive symptoms may accelerate the progression from mild cognitive impairment to dementia (Mourao et al., 2016). Previous studies have suggested that anxiety symptoms in individuals with mild cognitive impairment may serve as an early indicator of cognitive decline (Rozzini et al., 2009). Depressive symptoms has also been linked to greater atrophy in brain regions affected by Alzheimer’s disease. Among patients with depression, the annual conversion rate from mild cognitive impairment to dementia has been reported to range from 25.0% to 28.0% (Ma, 2020). Overall, anxiety and depressive symptoms may serve as important risk factors for cognitive decline and progression to dementia (Palmer et al., 2007).

Pharmacological treatment remains the primary approach for managing anxiety and depressive symptoms. However, it has several limitations, including slow onset, adverse effects, potential dependence and tolerance, and low adherence, which are more pronounced in older adults (Boerner, 2004; Song et al., 2023). Older patients often have multiple comorbidities and take several medications, increasing the risk of drug interactions. Therefore, relying solely on pharmacological treatment may not fully meet the clinical needs of older adults (Romdhani et al., 2023). Non-pharmacological interventions (NPIs) have received growing attention. NPIs refer to approaches that improve physical and mental health without medication through psychological, behavioral, physical, or environmental strategies (Bernardo-Filho et al., 2020). Psychotherapy is a first-line NPIs treatment for anxiety and depression (Cuijpers et al., 2016). However, its widespread implementation in primary community settings may be limited by the availability of trained professionals and healthcare resources (Wakida et al., 2018). Therefore, simple and accessible interventions, such as music therapy and physical exercise, may be more suitable for community-based implementation. A randomized controlled trial among older adults showed that resistance training improved anxiety and depressive symptoms, with within-group and between-group effect sizes of Cohen’s d = 0.38 and 0.78 for anxiety and 0.56 and 0.18 for depression, respectively (Carcelén-Fraile et al., 2024). Ramirez et al. reported that a novel neurofeedback-based music intervention improved depressive symptoms by 17.2% after 5 weeks of continuous exposure (Ramirez et al., 2015). However, anxiety and depression are complex and multifactorial conditions. Multidomain interventions targeting multiple risk factors and disease mechanisms may therefore provide greater preventive benefits. Compared with single-component interventions, multidomain NPIs involving two or more components may have greater effects on mental health in older adults. The Finnish Geriatric Intervention Study to Prevent Cognitive Impairment and Disability (FINGER) demonstrated that a multidomain intervention targeting modifiable risk factors could delay cognitive decline. This model has since been implemented and validated in several countries. The FINGER study also confirmed that multidomain lifestyle interventions are feasible, safe, and associated with high adherence (Ngandu et al., 2015). Nevertheless, evidence remains limited regarding the effectiveness of the multidomain NPIs for reducing anxiety and depressive symptoms in Chinese older adults with suspected cognitive impairment. Further research is warranted.

Anxiety and depressive symptoms are highly prevalent among older adults with cognitive impairment and substantially affect their quality of life. Therefore, this study conducted a 2-month multidomain NPIs among older adults with suspected cognitive impairment in Jiulongpo District, Chongqing, China. The aim was to evaluate the effects of this intervention on anxiety and depressive symptoms and provide evidence for clinical management in this population.

## 2 Methods

### 2.1 Study design and study procedure

#### 2.1.1 Participants and group allocation

This study was a community-based intervention conducted from September to December 2025 among older adults in Jiulongpo District, Chongqing, China. Doctors and staff from community health service centers screened participants’ cognitive function using the Ascertain Dementia 8-item Questionnaire (AD8) and the Community Screening Instrument for Dementia (CSI-D). Participants were classified as having suspected cognitive impairment if they tested positive on any one of the scales.

**FIGURE 1:**
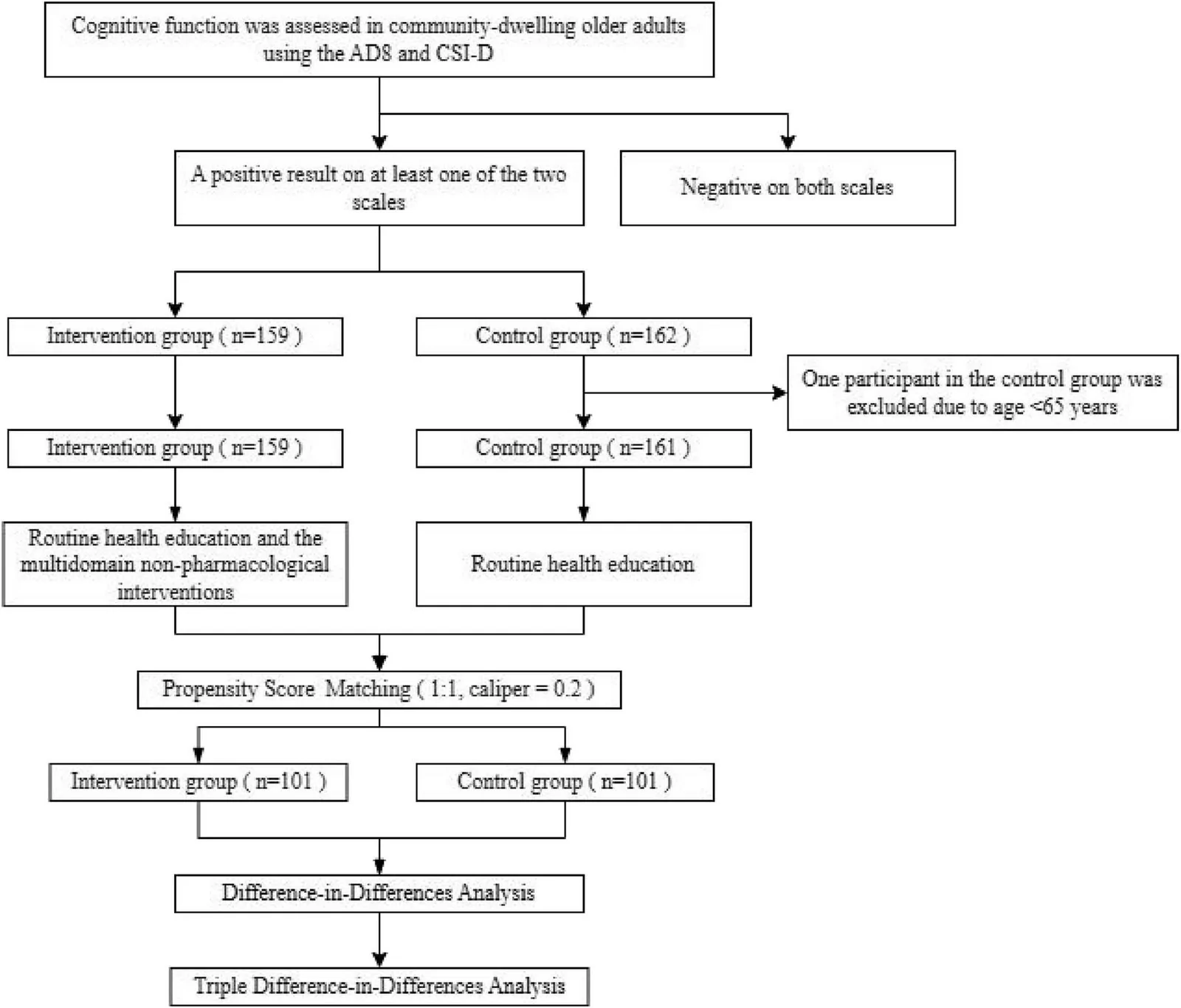
Flowchart of the study.

Participants were recruited through community announcements and study promotion during the cognitive function screening process. Eligible older adults who met the inclusion criteria and voluntarily agreed to participate were enrolled. To prevent contamination between groups within the same community and to ensure that the intervention could be delivered according to a standardized protocol, the intervention was implemented at the community health service center level. All participants from the same community health service center were assigned to the same study group. The selection of intervention and control sites considered factors such as geographic independence, population size served by each center, economic conditions, and service capacity. Ultimately, eight community health service centers implemented the intervention, while four centers served as control sites, thereby minimizing the potential influence of contextual heterogeneity on the interpretation of study findings. The inclusion criteria were as follows: (1) permanent residents of Jiulongpo District, Chongqing, China, aged 65 years or older; (2) classified as having suspected cognitive impairment; (3) able to communicate verbally; and (4) willing to participate and able to provide written informed consent. The exclusion criteria were as follows: (1) severe acute disease, end-stage chronic disease, or infectious disease; (2) long-term bedridden status or inability to stand independently; (3) receipt of cognitive function-targeted intervention within the past 30 days; and (4) being assessed as unsuitable for the intervention for safety reasons. A total of 321 participants were recruited, including 159 participants in the intervention group and 162 participants in the control group. One participant in the control group was excluded due to an age below 65 years. During the 2-month intervention period, participants received regular one-on-one communication to support attendance and ensure timely participation in intervention sessions. Small incentives were provided after completion of the intervention to encourage participant engagement and adherence. No participants withdrew from the study, resulting in a retention rate of 100%. All 159 participants in the intervention group completed all eight intervention sessions, with a completion rate of 100%. Finally, 159 participants in the intervention group and 161 participants in the control group were included in the final analysis. The detailed study flow is presented in Figure 1.

#### 2.1.2 Standardized training of research staff

To ensure the standardized and effective implementation of the intervention, all staff involved in intervention delivery received standardized training from the research team. The training included four main aspects: ➀ Pre-intervention training: Before the intervention began, all staff from participating community health service centers received centralized training. The research team demonstrated the complete procedures of each intervention session and developed standardized operating guidelines to ensure consistent implementation. ➁ Standardized materials: Standardized operating guidelines and intervention materials, including Baduanjin exercise videos and music audio files, were provided to all staff to ensure consistency in intervention content and delivery across different communities. ➂ On-site supervision: During the intervention period, the research team regularly visited community health service centers for on-site supervision. The implementation process was reviewed against the standardized procedures, and any deviations were identified and corrected promptly. ➃ Questionnaire administration training: Participants’ demographic characteristics and key study variables were collected through questionnaires. Before data collection, staff members received standardized training on questionnaire administration, and face-to-face interviews were conducted to collect information from participants, ensuring consistency and accuracy of data collection.

#### 2.1.3 The multidomain non-pharmacological interventions

The multidomain NPIs program was designed based on the Chinese Expert Consensus on Non-pharmacological Interventions for Cognitive Impairment Disorders (2025 edition) (Gong et al., 2026). The program included multiple components, such as music, physical exercise, and sensory stimulation. The intervention sessions were regularly delivered in the meeting rooms of community health service centers by at least two trained staff members from each center. The intervention lasted 2 months, with one session per week. A total of eight sessions were provided, and each session lasted 60–90 min.

The control group received routine health education. Health education was delivered through community bulletin boards, health brochures, WeChat official accounts, and other media platforms. The main content covered brain health and dementia prevention and management. In addition to routine health education, the intervention group received the multidomain NPIs. The detailed intervention content is shown in Table 1.

**TABLE 1:** Components of the multidomain non-pharmacological interventions program.

Week	Intervention	Intervention content
Week 1	Singing together as a group	Familiar song excerpts were played, and participants were invited to identify the song titles based on the melodies. Subsequently, with guidance from the staff, participants sang the complete songs together with other group members to promote reminiscence, social interaction, and emotional engagement.
Week 2	Reminiscence of classic films	Participants were asked to sequentially recall the movies they had watched, including the locations of viewing and their favorite films. This was followed by group viewing of selected classic movie clips to stimulate memory and discussion.
Week 3	Baduanjin exercise	Under safety supervision, participants practiced the traditional Chinese health exercise “Baduanjin,” which aims to improve physical fitness and enhance sleep quality.
Week 4	Card games	1. Staff instructed participants in hand gestures representing numbers and arithmetic symbols, followed by exercises in performing addition and subtraction using these gestures. Each participant completed three problems. 2. Several cards were randomly placed on a table, and participants were asked to memorize their positions, colors, and sizes. After the cards were flipped, participants were required to identify specified cards, aiming to train memory and attention.
Week 5	Spatiotemporal perception training	1. Staff gave simple motor commands, such as “raise your right hand” or “touch your left ear”, and participants were asked to respond accordingly. This activity was designed to train attention, comprehension, and motor coordination. 2. Two ropes or floor markings were used to set a fixed distance. Participants were asked to estimate the number of steps required to walk the distance, and their estimates were then compared with the actual number of steps taken. This activity aimed to enhance spatial judgment and body awareness.
Week 6	Simulated shopping and payment activity	Staff organized simulated buying-and-selling activities based on daily-life scenarios. Participants were asked to select and combine at least two payment methods according to the price of each item, aiming to train calculation skills, problem-solving ability, and daily living competence.
Week 7	Art class	Participants were provided with paint-by-numbers kits or paper-cutting materials and were guided to complete painting or window paper-cutting activities. This activity was designed to train fine motor skills, attention, and hands-on ability.
Week 8	Memory journey	1. Participants were asked to bring personal photo albums or photographs and to recall and share the stories behind the pictures within the group. This activity was intended to promote autobiographical memory, verbal expression, and social interaction. 2. Participants were guided to review the main activities completed during the previous eight sessions, helping them summarize their intervention experiences and reinforce their sense of participation.

#### 2.1.4 Ethical approval and informed consent

This study was conducted in accordance with the principles of the Declaration of Helsinki and was approved by the Ethics Committee of the Disease Control and Prevention Center of Jiulongpo District, Chongqing, China (Protocol code: JLPJK-KX-2024-02). Written informed consent was obtained from all participants prior to the intervention.

### 2.2 Ascertain dementia 8-item questionnaire and community screening instrument for dementia

AD8 and CSI-D are widely validated cognitive impairment screening tools for community settings. In Chinese populations, AD8 showed good diagnostic performance, with an optimal cutoff value of 2 for distinguishing cognitively normal individuals from those with dementia (AUC = 0.961, sensitivity = 97.6%, specificity = 78.1%) (Yang et al., 2011). Cross-cultural validation studies of CSI-D have reported a sensitivity of approximately 87% and a specificity of approximately 83%, and the Chinese version has also demonstrated good reliability and validity (Liu et al., 2005; Prince et al., 2003). The combined use of AD8 and CSI-D has been recommended in resource-limited community settings to improve screening accuracy through complementary advantages.

The AD8 was used to identify subjective cognitive decline (Galvin et al., 2005). It includes eight items: impaired judgment, reduced interest in hobbies or activities, repeating the same things, difficulty learning to use tools or appliances, forgetting the correct month or year, difficulty managing complex financial affairs, difficulty remembering appointments, and daily problems with thinking or memory. Each item rated as “hanged” is scored as 1 point. The total score ranges from 0 to 8. Higher scores indicate poorer cognitive function. A total score of ≥2 was used to define suspected cognitive impairment (Galvin et al., 2006).

The CSI-D includes seven tasks: naming the elbow, identifying the use of a hammer, locating the nearest market or shop, identifying the day of the week, identifying the season, pointing first to the window and then to the door, and immediate and delayed recall of three words (Chan et al., 2003). Each correct response is scored as 1 point. The total score ranges from 0 to 9. A total score of ≤7 was used to define suspected cognitive impairment (Prince et al., 2011).

### 2.3 Assessments of anxiety symptoms

Anxiety symptoms were assessed using the Generalized Anxiety Disorder Scale (GAD-7). The scale measures generalized anxiety symptoms over the past 2 weeks. It includes seven items. Each item is rated on a 4-point Likert scale: 0 = “not at all”, 1 = “several days”, 2 = “more than half the days”, and 3 = “nearly every day”. The total GAD-7 score ranges from 0 to 21. Higher scores indicate more severe anxiety symptoms. A total score of ≥5 was used to indicate the presence of anxiety symptoms (Spitzer et al., 2006).

### 2.4 Assessments of depressive symptoms

The 30-item Geriatric Depression Scale (GDS) was developed by Brink and Yesavage in 1982 (Yesavage et al., 1982). Based on this scale, Sheikh and Yesavage developed a shorter version in 1986, the 15-item Geriatric Depression Scale (GDS-15) (Cruice et al., 2011). The GDS-15 is simple to operate and requires little time. Therefore, it has been widely used in psychological studies of older adults in China . The scale assesses depressive symptoms over the past week. It contains 15 items, including 5 reverse-scored items. The total score ranges from 0 to 15. Higher scores indicate more severe depressive symptoms. A total GDS-15 score of ≥8 was used to indicate the presence of depressive symptoms (Parsons et al., 2024).

### 2.5 Covariates

A total of 11 covariates were adjusted for in the model, covering three domains: sociodemographic characteristics, behavioral factors, and health-related factors. Sociodemographic characteristics included gender, age, education level, marital status, and living arrangement. Behavioral factors included walking, smoking, and drinking. Health-related factors included hypertension, diabetes, and cognitive status.

Cognitive status was assessed using the Mini-Mental State Examination (MMSE), a widely used tool for cognitive evaluation. The MMSE covers orientation, memory, attention and calculation, recall, and language ability, comprising 30 items. Each correct response is scored 1 point, and each incorrect response is scored 0, giving a total score of 30. Cognitively impaired was defined using education-specific cut-off scores: MMSE ≤17 for illiterate participants, ≤20 for those with primary school education, and ≤ 24 for those with junior middle school education or higher (Su et al., 2014).

### 2.6 Statistical analysis

Anxiety and depressive symptoms were the primary outcomes of this study. A two-sided test was used, with α = 0.05,β = 0.20,Z_1–α/2_ = 1.96,Z_1–β_ = 0.84. In the formula, δ represents the difference in means between the two groups, and σ denotes the pooled standard deviation. The sample size was calculated using the following formula (Clifton et al., 2019):


$$n=\frac{2\,{\left(Z_{1-\alpha /2}+Z_{1-\beta }\right)}^{2}\,\sigma^{2}}{\delta^{2}}$$

$$\sigma =\sqrt{\frac{\left(n_{1}-1\right)\,s_{1}^{2}+\left(n_{2}-1\right)\,s_{2}^{2}}{n_{1}+n_{2}-2}}$$

Based on previous studies, the sample size estimated using depressive symptoms as the outcome measure was relatively small (Eimontas et al., 2025; Xue et al., 2023). Therefore, the sample size calculated based on anxiety symptoms was adopted as the final sample size. According to the findings reported by Eimontas et al., the estimated effect size and standard deviation were 4.66 and 2.06, respectively, resulting in a required sample size of 81 participants per group. Considering a 10% loss to follow-up, at least 90 participants were needed in both the intervention and control groups.

The normality of continuous variables was assessed using the Kolmogorov–Smirnov test. Variables with *P*-values less than 0.05 were considered non-normally distributed. Continuous variables that did not follow a normal distribution were summarized as median and interquartile range [M (Q_1_, Q_3_)], whereas categorical variables were expressed as frequency and percentage [n (%)]. Baseline characteristics of the intervention and control groups were compared using the Wilcoxon test and chi-square test.

Propensity Score Matching (PSM) was applied to balance baseline characteristics between the intervention and control groups. Matching was performed using a 1:1 nearest neighbor algorithm with a caliper width of 0.2 standard deviations (Yuan et al., 2026). The matching covariates included AD8 scores, CSI-D scores, gender, age, education level, marital status, living arrangement, walking, smoking, drinking, hypertension, diabetes, cognitive status, baseline anxiety and depressive symptom scores (Brookhart et al., 2006). Standardized mean differences (SMD) were used to evaluate baseline balance, with an absolute SMD <0.10 indicating satisfactory balance. The effectiveness of matching was visualized using propensity score density plots and Love plots.

The Difference-in-Differences (DID) approach was employed to address endogeneity arising from self-selection bias and omitted variables (Phng et al., 2024). As a quasi-experimental design commonly used for causal inference in public health research, DID enables the estimation of intervention effects attributable to net effects (Wing et al., 2018). The DID model was specified as follows:


$$\text{Y}=\beta_{0}+\beta_{1}\,\text{treat}+\beta_{2}\,\text{time}+\beta_{3}\,\left(\text{treat }\times \text{time}\right)+\gamma \,\text{X}+\varepsilon$$

Y represents the scores of depressive or anxiety symptoms; treat indicates group assignment, including the intervention and control groups, with the control group coded as 0 and the intervention group coded as 1; time represents the measurement time point, including pre-intervention and post-intervention assessments, with pre-intervention coded as 0 and post-intervention coded as 1; the interaction term (treat × time) captures the intervention effect; X includes covariates for adjustment; and ε is the random error.

To evaluate the effects of the multidomain NPIs on anxiety and depressive symptoms in older adults with suspected cognitive impairment, three models were constructed using robust standard errors clustered by PSM-matched pair identifiers (Su et al., 2025): Model 1 was unadjusted; Model 2 controlled for sociodemographic characteristics, including gender, age, education level, marital status, and living arrangement; Model 3 further adjusted for behavioral and health characteristics, including walking, smoking, drinking, hypertension, diabetes, and cognitive status.

Furthermore, a Difference-in-Difference-in-Differences (DDD) model was constructed by incorporating baseline cognitive status into the DID framework. This model was used to assess whether cognitive status moderated the effects of the multidomain NPIs on anxiety and depressive symptoms, thereby examining heterogeneity in intervention effects across different cognitive status.

All statistical tests were two-sided, and a *P*-value of < 0.05 was considered statistically significant. Statistical analyses were conducted using SPSS version 27.0 and R version 4.3.

## 3 Results

### 3.1 Characteristics among participants

Among individuals with suspected cognitive impairment, the prevalence of anxiety and depressive symptoms was 24.38% and 10.94%, respectively. The baseline AD8 scores were 3.00 in the intervention group and 2.00 in the control group, while the corresponding baseline CSI-D scores were 8.00 and 7.00, respectively.

As shown in Table 2, there were no significant baseline differences between the intervention and control groups in gender, age, walking, smoking, drinking, hypertension, diabetes, anxiety symptoms, cognitive status (binary) or CSI-D scores (*P* > 0.05). These results indicated good comparability for these variables. Notably, diabetes status showed a borderline difference between the two groups (*P* = 0.051), suggesting a potential baseline difference. This potential imbalance will be carefully considered in subsequent analyses. Significant differences were observed in living arrangement, marital status, educational level, depressive symptoms, and AD8 scores (*P* < 0.05). Therefore, PSM was used to balance baseline characteristics between the two groups. This approach improved group comparability and strengthened the validity and robustness of the subsequent DID regression analyses.

**TABLE 2:** Baseline characteristics of the intervention and control groups before matching.

Variables	Total	Control	Intervention	Statistic	*P*
Gender				χ^2^ = 2.10	0.147
Male	110 (34.38%)	62 (38.51%)	48 (30.19%)		
Female	210 (65.63%)	99 (61.49%)	111 (69.81%)		
Age				χ^2^ = 1.29	0.256
65–80 years	208 (65.00%)	110 (68.32%)	98 (61.64%)		
≥80 years	112 (35.00%)	51 (31.68%)	61 (38.36%)		
Living arrangement				χ^2^ = 7.02	0.008
Living with spouse	226 (70.63%)	125 (77.64%)	101 (63.52%)		
Others	94 (29.38%)	36 (22.36%)	58 (36.48%)		
Marital status				χ^2^ = 9.27	0.002
Married	229 (71.56%)	128 (79.50%)	101 (63.52%)		
Others	91 (28.44%)	33 (20.50%)	58 (36.48%)		
Education level				χ^2^ = 17.49	<0.001
Primary school and below	152 (47.50%)	58 (36.02%)	94 (59.12%)		
Junior high school	93 (29.06%)	59 (36.65%)	34 (21.38%)		
High school / technical school or above	75 (23.44%)	44 (27.33%)	31 (19.50%)		
Walking				χ^2^ = 0.30	0.584
No	135 (42.19%)	65 (40.37%)	70 (44.03%)		
Yes	185 (57.81%)	96 (59.63%)	89 (55.97%)		
Smoking				χ^2^ = 0.00	1.000
No	295 (92.19%)	148 (91.93%)	147 (92.45%)		
Yes	25 (7.81%)	13 (8.07%)	12 (7.55%)		
Drinking				χ^2^ = 0.97	0.325
No	281 (87.81%)	138 (85.71%)	143 (89.94%)		
Yes	39 (12.19%)	23 (14.29%)	16 (10.06%)		
Hypertension				χ^2^ = 0.44	0.505
No	168 (52.50%)	88 (54.66%)	80 (50.31%)		
Yes	152 (47.50%)	73 (45.34%)	79 (49.69%)		
Diabetes				χ^2^ = 3.81	0.051
No	216 (67.50%)	100 (62.11%)	116 (72.96%)		
Yes	104 (32.50%)	61 (37.89%)	43 (27.04%)		
Cognitive Status (Binary)				χ^2^ = 0.04	0.843
Without objective cognitive impairment	210 (65.63%)	107 (66.46%)	103 (64.78%)		
Cognitively impaired	110 (34.38%)	54 (33.54%)	56 (35.22%)		
Depression symptoms (Binary)				χ^2^ = 18.14	<0.001
No	285 (89.06%)	131 (81.37%)	154 (96.86%)		
Yes	35 (10.94%)	30 (18.63%)	5 (3.14%)		
Anxiety symptoms (Binary)				χ^2^ = 3.57	0.059
No	242 (75.63%)	114 (70.81%)	128 (80.50%)		
Yes	78 (24.38%)	47 (29.19%)	31 (19.50%)		
Cognitive Status	25.00 (21.00, 28.00)	25.00 (22.00, 28.00)	25.00 (19.00, 27.00)	Z = 14515.5	0.038
Depression symptoms	3.00 (1.00, 5.00)	3.00 (1.00, 6.00)	2.00 (1.00, 4.00)	Z = 15932.50	<0.001
Anxiety symptoms	0.00 (0.00, 4.00)	0.00 (0.00, 5.00)	0.00 (0.00, 4.00)	Z = 13579.50	0.307
AD8	2.00 (1.00, 4.00)	2.00 (1.00, 2.00)	3.00 (2.00, 4.00)	Z = 8422	<0.001
CSI-D	7.00 (7.00, 8.00)	7.00 (7.00, 8.00)	8.00 (7.00, 9.00)	Z = 12011	0.327

χ^2^: Chi-square test; Z: Wilcoxon rank sum test AD8: Ascertain Dementia 8-item Questionnaire; CSI-D: Community Screening Instrument for Dementia.

### 3.2 Propensity score matching results

After 1:1 PSM was performed to minimize baseline differences, 101 participants were retained in the intervention group and 101 in the control group, yielding a final matched sample of 202 participants. After matching, the SMD for all baseline characteristics were below 0.1, indicating satisfactory covariate balance. No statistically significant between-group differences were observed for baseline variable after matching (*P* > 0.05). Detailed results are presented in Supplementary Figure 1 and Supplementary Table 1.

**FIGURE 2:**
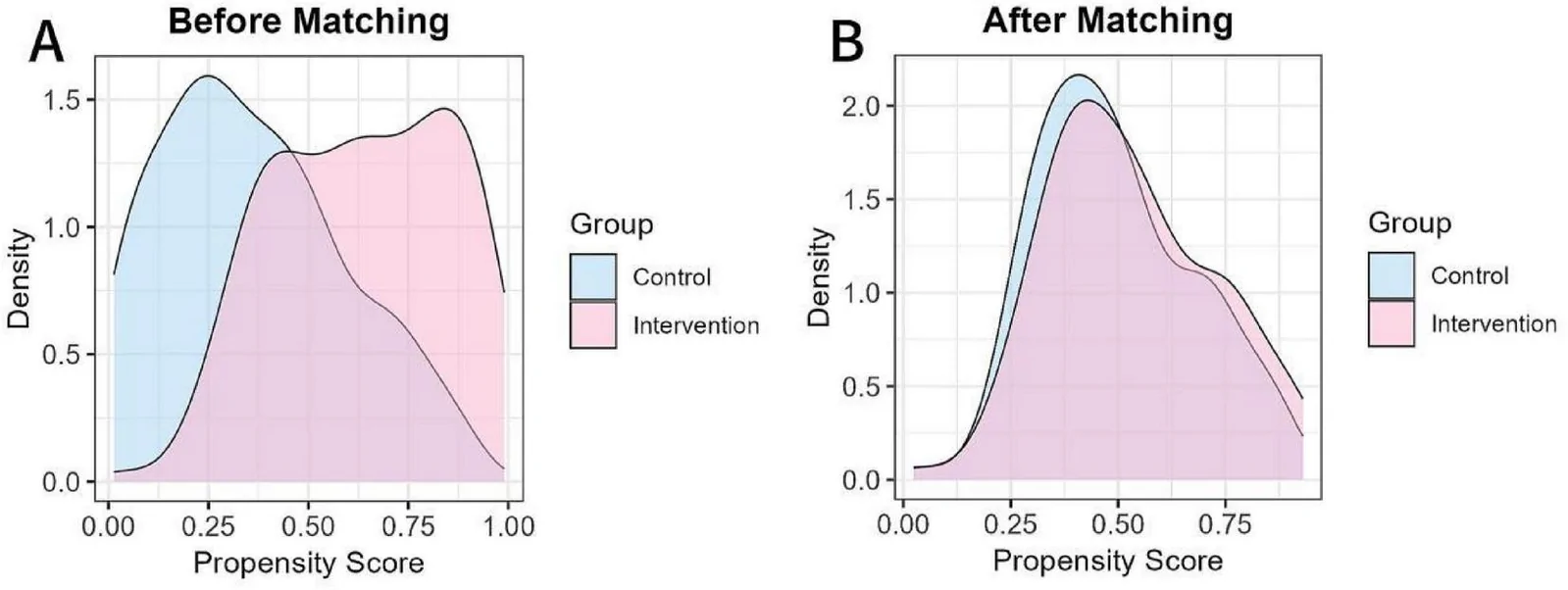
Propensity score distributions of control and intervention groups before and after matching. **(A)** Before matching. **(B)** After matching.

The propensity score density plot was used to visually assess covariate balance between the intervention and control groups before and after matching. Before PSM, the density curves showed limited overlap between the two groups, suggesting imbalance in baseline covariates (Figure 2A). After PSM, the density curves of the two groups overlapped closely and showed highly similar distribution patterns (Figure 2B), indicating improved baseline comparability between the groups.

### 3.3 Difference-in-differences analysis of anxiety symptoms and depressive symptoms after the multidomain non-pharmacological interventions.

As shown in Table 3, the multidomain NPIs significantly reduced anxiety and depressive symptoms among older adults with suspected cognitive impairment. Specifically, in the three progressively adjusted models, depressive symptom scores increased by 1.48 points over time (Model 1: 95% CI: 0.79, 2.16; Model 2: 95% CI: 0.79, 2.16; Model 3: 95% CI: 0.78, 2.17). The “Treatment × Time” interaction term was statistically significant in all three models. Compared with the control group, the intervention group exhibited a reduction of 2.04 points in depressive symptom scores (Model 1: 95% CI: −2.81, −1.26; Model 2: 95% CI: −2.82, −1.26; Model 3: 95% CI: −2.83, −1.25).

**TABLE 3:** Difference-in-differences estimates of the effects of the multidomain non-pharmacological interventions on anxiety and depressive symptoms.

Outcome	Model	Variable	β (95% CI)	*P*
Depressive Symptoms	Model 1	Treatment	0.01 (−0.64, 0.66)	0.976
		Time	1.48 (0.79, 2.16)	<0.001
		Treatment × Time	−2.04 (−2.81, −1.26)	<0.001
	Model 2	Treatment	−0.15 (−0.72, 0.42)	0.599
		Time	1.48 (0.79, 2.16)	<0.001
		Treatment × Time	−2.04 (−2.82, −1.26)	<0.001
	Model 3	Treatment	−0.11 (−0.59, 0.37)	0.650
		Time	1.48 (0.78, 2.17)	<0.001
		Treatment × Time	−2.04 (−2.83, −1.25)	<0.001
Anxiety Symptoms	Model 1	Treatment	−0.07 (−0.90, 0.76)	0.870
		Time	0.27 (−0.32, 0.85)	0.371
		Treatment × Time	−1.44 (−2.21, −0.66)	<0.001
	Model 2	Treatment	−0.19 (−1.01, 0.62)	0.641
		Time	0.27 (−0.32, 0.86)	0.374
		Treatment × Time	−1.44 (−2.22, −0.65)	<0.001
	Model 3	Treatment	−0.15 (−0.93, 0.63)	0.710
		Time	0.27 (−0.33, 0.86)	0.378
		Treatment × Time	−1.44 (−2.22, −0.65)	<0.001

Model 1 unadjusted model. Model 2 controlling for gender, age, living arrangement, marital status, education level. Model 3 controlling for gender, age, living arrangement, marital status, education level, walking, smoking, drinking, hypertension, diabetes and cognitive status.

For anxiety symptoms, no significant time trend was observed. However, the “Treatment × Time” interaction indicated a significant decrease in anxiety scores in the intervention group across all three models, with scores 1.44 points lower than those of the control group (Model 1: 95% CI: −2.21, −0.66; Model 2: 95% CI: −2.22, −0.65; Model 3: 95% CI: −2.22, −0.65).

### 3.4 Effect modification by baseline cognitive status

Building on the DID framework, a DDD model was constructed to examine whether baseline cognitive status moderated the effects of the multidomain NPIs on anxiety and depressive symptoms. Based on MMSE scores, participants were categorized into without objective cognitive impairment and cognitively impaired groups. Three models were specified: Model 1 was unadjusted; Model 2 adjusted for gender, age, education level, marital status, and living arrangement; and Model 3 further adjusted for walking, smoking, alcohol consumption, hypertension, and diabetes.

For depressive symptoms, the triple interaction term was statistically significant across all three models. In the fully adjusted Model 3, the multidomain NPIs was associated with a significant reduction in depressive symptom scores (β = −2.75, 95% CI: −3.67, −1.84, *P* < 0.001). Baseline cognitive status significantly modified the effect of the multidomain NPIs on depressive symptoms (Treatment × Time × Cognitive status: β = 2.14, 95% CI: 0.25-4.04, *P* = 0.027). Specifically, the multidomain NPIs was associated with an average reduction of 2.75 points in depressive symptom scores among older adults without objective cognitive impairment at baseline. In contrast, the reduction was only 0.61 points among those with baseline cognitive impairment. These findings suggest that, compared with older adults with baseline cognitive impairment, the intervention showed a greater effect on reducing depressive symptoms among older adults without objective cognitive impairment at baseline.

For anxiety symptoms, the triple-interaction term was not statistically significant in any of the three models (Model 1: 95% CI: −0.06, 3.6, *P* = 0.058; Model 2: 95% CI: −0.07, 3.61, *P* = 0.06; Model 3: 95% CI: −0.08, 3.62, *P* = 0.061), indicating insufficient evidence that baseline cognitive status modified the effect of the intervention on anxiety symptoms. Effect estimates remained largely unchanged after adjustment for covariates, supporting the robustness of these findings. Detailed results are presented in Table 4.

**TABLE 4:** Triple difference-in-differences estimates for the effect modification by baseline cognitive status on depressive and anxiety symptoms.

Outcome	Model	Variables	β (95% CI)	*P*-value
Depressive symptoms	Model1	Treatment × Time	−2.75 (−3.66, −1.85)	< 0.001
		Treatment × Cognitive status	−2.87 (−4.58, −1.17)	0.001
		Time × Cognitive status	−2.00 (−3.58, −0.42)	0.013
		Treatment × Time × Cognitive status	2.14 (0.27, 4.01)	0.026
	Model2	Treatment × Time	−2.75 (−3.66, −1.85)	<0.001
		Treatment × Cognitive status	−2.57 (−4.13, −1.00)	0.001
		Time × Cognitive status	−2.00 (−3.59, -0.41)	0.014
		Treatment × Time × Cognitive status	2.14 (0.26, 4.02)	0.026
	Model3	Treatment × Time	−2.75 (−3.67, −1.84)	<0.001
		Treatment × Cognitive status	−2.63 (−4.19, −1.08)	0.001
		Time × Cognitive status	−2.00 (−3.60, −0.40)	0.015
		Treatment × Time × Cognitive status	2.14 (0.25, 4.04)	0.027
Anxiety symptoms	Model1	Treatment × Time	−2.04 (−2.84, −1.24)	<0.001
		Treatment × Cognitive status	−3.71 (−5.51, −1.91)	<0.001
		Time × Cognitive status	−1.91 (−3.32, −0.50)	0.008
		Treatment × Time × Cognitive status	1.77 (−0.06, 3.60)	0.058
	Model2	Treatment × Time	−2.04 (−2.84, −1.23)	<0.001
		Treatment × Cognitive status	−3.54 (−5.25, −1.83)	<0.001
		Time × Cognitive status	−1.91 (−3.33, −0.49)	0.009
		Treatment × Time × Cognitive status	1.77 (−0.07, 3.61)	0.06
	Model3	Treatment × Time	−2.04 (−2.85, −1.23)	<0.001
		Treatment × Cognitive status	−3.73 (−5.37, −2.10)	<0.001
		Time × Cognitive status	−1.91 (−3.34, −0.48)	0.009
		Treatment × Time × Cognitive status	1.77 (−0.08, 3.62)	0.061

Model 1 unadjusted model. Model 2 controlling for gender, age, living arrangement, marital status, education level. Model 3 controlling for gender, age, living arrangement, marital status, education level, walking, smoking, drinking, hypertension and diabetes.

## 4 Discussion

This study focused on older adults with suspected cognitive impairment and employed PSM combined with a DID approach to evaluate the effects of the multidomain NPIs on anxiety and depressive symptoms. The results showed that the multidomain NPIs significantly reduced anxiety symptoms (β = −1.44, 95% CI: −2.22, −0.65) and depressive symptoms (β = −2.04, 95% CI: −2.83, −1.25) in this population. Further DDD analyses indicated that baseline cognitive status may modify the effect of the multidomain NPIs on depressive symptom improvement. Specifically, compared with older adults with baseline cognitive impairment, the intervention showed a greater effect on reducing depressive symptoms among older adults without objective cognitive impairment at baseline.

The present study found that the prevalence of anxiety and depressive symptoms among older adults with suspected cognitive impairment in China was 24.38% and 10.94%, respectively. These findings are broadly consistent with previous studies (Chen et al., 2018; Ismail et al., 2017). Minor differences across studies may be attributable to variations in measurement instruments, study regions, and national contexts. Several neurobiological mechanisms have been proposed to explain anxiety and depressive symptoms associated with cognitive decline in older adults. Cognitive decline may affect emotional regulation through alterations in specific brain regions and neuroendocrine pathways. First, the dorsolateral prefrontal cortex (DLPFC) plays a critical role in executive function and emotion regulation. Cognitive decline is often accompanied by reduced DLPFC gray matter and impaired function, which may weaken the ability to regulate negative emotions. As a result, daily stressors may continuously activate emotional circuits, increasing the risk of anxiety and depressive symptoms (Mayberg, 1997). Second, the prefrontal cortex regulates the amygdala through top-down inhibitory control (Disner et al., 2011). Cognitive decline may involve reduced prefrontal gray matter volume and impaired white matter integrity, leading to weakened inhibitory regulation. The resulting amygdala hyperactivity may contribute to abnormal activation of fear and anxiety-related circuits. Animal studies further support that dysfunction of the prefrontal-amygdala circuit is associated with anxiety-like behaviors (Liu et al., 2020). Third, hippocampal alterations associated with cognitive decline may disrupt hypothalamic-pituitary-adrenal (HPA) axis regulation (Sapolsky, 2000). Reduced hippocampal glucocorticoid receptor expression may impair negative feedback control, resulting in persistent HPA axis activation and elevated glucocorticoid levels. This process may further affect serotonergic function and increase the risk of depression.

The multidomain NPIs in this study was designed to target multiple domains, including physical exercise, music, cognitive training, and spatial perception. DID analyses showed that these interventions significantly reduced anxiety and depressive symptom among older adults with suspected cognitive impairment, consistent with previous findings. A meta-analysis has reported that physical exercise alleviates depressive and anxiety symptoms, with aerobic exercise showing the most pronounced effects (Munro et al., 2026). From a physiological perspective, exercise-related interventions may exert antidepressant and anxiolytic effects partly through brain-derived neurotrophic factor (BDNF). BDNF is a protein found in the central nervous system. It plays an important role in neural plasticity, synapse formation, and neuronal survival. Regular aerobic exercise can increase BDNF levels, which may mediate the beneficial effects of exercise on emotional states (Huang et al., 2014). Music interventions have also been found to relieve anxiety and depressive symptoms in patients with dementia. Zhang and colleagues summarized previous studies, indicating that short interventions lasting over 45 min once per week are particularly effective for emotion regulation (Zhang et al., 2023). A 2021 meta-analysis of 32 randomized controlled trials, including 1,924 participants, further demonstrated that music therapy significantly reduced anxiety symptoms during treatment (Lu et al., 2021). The underlying mechanism may involve modulation of autonomic nervous system function. Calm and rhythmically regular music can enhance parasympathetic activity and reduce sympathetic excitability, thereby attenuating stress responses (Bernardi et al., 2006). In addition, music-induced pleasure may involve activation of the mesolimbic dopaminergic rewards pathway, which is important for emotion regulation, motivation, and positive affect, thereby improving psychological well-being (Koelsch, 2014). The observed benefits in this study suggest that improvements in anxiety and depressive symptoms may be achieved through multiple synergistic pathways. The FINGER study demonstrated that multidomain interventions can jointly enhance BDNF regulation and preserve telomere length, with effects beyond the simple addition of individual interventions (Ngandu et al., 2015). This multidomain NPIs may simultaneously target neuroplasticity, neuroendocrine regulation, neuroinflammation, and social-related pathways, leading to synergistic effects. In addition, the multidomain NPIs in our study was primarily delivered through group-based activities. According to the Social Identity Approach to Health, group activities can strengthen social connections, foster a sense of belonging and identity, and provide emotional support and social resources, thereby buffering psychological stress and promoting mental health (Hopkins and Reicher, 2016). For older adults, group activities may also reduce feelings of loneliness and social isolation while enhancing engagement and perceived self-worth. Previous studies by Quinn and Francis similarly reported that group-based interventions reduce anxiety and depressive symptoms among older adults (Francis et al., 2022; Quinn et al., 2025).

DDD analysis further indicated that baseline cognitive status moderated the intervention effect on depressive symptoms, but not on anxiety symptoms. This may be related to differences in the neurobiological mechanisms of depression and anxiety. For depressive symptoms, participants without objective cognitive impairment appeared to benefit more from the multidomain NPIs than the cognitively impaired group. These findings suggest that implementing interventions before significant cognitive impairment develops may lead to greater benefits, providing support for earlier intervention strategies in community settings. Improvement in depressive symptoms may rely on higher-order cognitive processes related to prefrontal cortex function. The prefrontal-amygdala circuit is central to emotion regulation and cognitive integration (Challis and Berton, 2015). In individuals with cognitive impairment, prefrontal cortical atrophy, weakened functional connectivity, and reduced executive function may limit their ability to regulate negative emotions, resulting in smaller benefits from the multidomain NPIs (Fales et al., 2008). Compared with depressive symptoms, anxiety symptoms are more closely associated with physiological arousal of the autonomic nervous system (Shin and Liberzon, 2010). The multidomain NPIs may relieve anxiety-related symptoms by reducing sympathetic nervous system activity, enhancing parasympathetic activity, and attenuating stress responses (Anderson and Shivakumar, 2013). This process may rely less on higher-order cognitive function.

The findings of this study provide evidence to inform psychological health interventions for older adults with suspected cognitive impairment in China. First, compared with routine health education, participation in the multidomain NPIs was associated with reduced anxiety and depressive symptoms among older adults with suspected cognitive impairment. These findings suggest that integrating such interventions into community-based cognitive impairment management may help improve emotional health outcomes in this population. Second, the results suggest that primary care institutions, such as community health centers and eldercare facilities, can develop targeted intervention programs based on the severity of cognitive impairment. Implementing flexible the multidomain NPIs may enhance effectiveness and optimize resource use. Third, compared with single-component interventions, multidomain interventions usually require greater resource investment, higher participant engagement, and more support from trained professionals. However, the intervention in this study was delivered by community health workers who received standardized training, reducing reliance on external professionals. The intervention was delivered in community health service centers and used low-cost, easily accessible materials, which further enhanced its feasibility and potential for broader implementation. The standardized training protocol also improved the potential for replication across different community settings. However, future implementation efforts should carefully consider factors such as maintaining stable intervention venues, ensuring continuous staff support, and promoting long-term participant engagement.

This study has several limitations. First, this study used a non-randomized design and may therefore be subject to potential confounding due to baseline differences. Although PSM achieved good balance in baseline covariates between the groups, residual confounding from unmeasured factors, such as household economic status, social support, and health literacy, cannot be excluded. Second, while the overall sample size met the minimum requirement, the number of participants in different cognitive status subgroups was relatively small, which may reduce the precision of effect estimates. Future studies should include larger samples to validate these findings and allow more detailed subgroup analyses. Third, this study did not differentiate between primary and secondary causes of cognitive impairment. Therefore, some individuals with suspected cognitive impairment may have cognitive symptoms primarily related to depression or anxiety disorders rather than neurodegenerative processes. Although AD8 and CSI-D were used as complementary screening tools and strict exclusion criteria were applied, the lack of clinical differential diagnosis remains an important limitation. Future studies will need to incorporate clinical diagnoses and more comprehensive neuropsychological assessments to better characterize the nature of cognitive impairment. Finally, this was a single-center study, and all participants were recruited from the same region, which may limit the generalizability of the results. Multicenter and cross-regional studies are warranted to further evaluate the applicability and effectiveness of the multidomain NPIs in diverse populations.

## 5 Conclusion

The results showed that the multidomain NPIs may moderately reduce anxiety and depressive symptoms in older adults with suspected cognitive impairment. Further DDD analysis showed that the effect on depressive symptoms may be influenced by baseline cognitive status. Specifically, compared with older adults with baseline cognitive impairment, the multidomain NPIs showed a greater effect on reducing depressive symptoms among older adults without objective cognitive impairment at baseline. The multidomain NPIs used in this study included multiple components and showed good comprehensiveness and feasibility. Integrating the multidomain NPIs into the early management of cognitive impairment in older adults may contribute to better emotional health outcomes.

## Data Availability

The raw data supporting the conclusions of this article will be made available by the authors, without undue reservation.
