# Supplementary material for: Effects of the multidomain non-pharmacological interventions on anxiety symptoms and depressive symptoms in older adults with suspected cognitive impairment: a propensity score matching and difference-in-differences analysis
Source: Front Aging Neurosci. 2026 Aug 21;18:1921356. doi: 10.3389/fnagi.2026.1921356 (PMC13541682; doi:10.3389/fnagi.2026.1921356)
Supplement: Supplementary file 1 [file Data_Sheet_1.pdf]

Supplementary Material

Table S1. Baseline characteristics of the intervention and control groups after matching.

Variables	Total	Control	Intervention	Statistic	*P*
**Gender**				$\chi^2 = 0.02$	0.883
Male	72 (35.64%)	37 (36.63%)	35 (34.65%)		
Female	130 (64.36%)	64 (63.37%)	66 (65.35%)		
**Age**				$\chi^2 = 0.53$	0.465
65 - 80 years	128 (63.37%)	67 (66.34%)	61 (60.40%)		
≥80 years	74 (36.63%)	34 (33.66%)	40 (39.60%)		
**Living arrangement**				$\chi^2 = 0.59$	0.441
Living with spouse	142 (70.30%)	74 (73.27%)	68 (67.33%)		
Others	60 (29.70%)	27 (26.73%)	33 (32.67%)		
**Marital status**				$\chi^2 = 0.23$	0.633
Married	148 (73.27%)	76 (75.25%)	72 (71.29%)		
Others	54 (26.73%)	25 (24.75%)	29 (28.71%)		
**Education level**				$\chi^2 = 2.11$	0.348
Primary school and below	89 (44.06%)	40 (39.60%)	49 (48.51%)		
Junior high school	59 (29.21%)	30 (29.70%)	29 (28.71%)		

High school / technical school or above	54 (26.73%)	31 (30.69%)	23 (22.77%)		
**Walking**				$\chi^2 = 0$	1.000
No	79 (39.11%)	40 (39.60%)	39 (38.61%)		
Yes	123 (60.89%)	61 (60.40%)	62 (61.39%)		
**Smoking**				$\chi^2 = 0$	1.000
No	186 (92.08%)	93 (92.08%)	93 (92.08%)		
Yes	16 (7.92%)	8 (7.92%)	8 (7.92%)		
**Drinking**				$\chi^2 = 0.04$	0.843
No	172 (85.15%)	85 (84.16%)	87 (86.14%)		
Yes	30 (14.85%)	16 (15.84%)	14 (13.86%)		
**Hypertension**				$\chi^2 = 0.18$	0.673
No	102 (50.50%)	53 (52.48%)	49 (48.51%)		
Yes	100 (49.50%)	48 (47.52%)	52 (51.49%)		
**Diabetes**				$\chi^2 = 0$	1.000
No	141 (69.80%)	70 (69.31%)	71 (70.30%)		
Yes	61 (30.20%)	31 (30.69%)	30 (29.70%)		
**Cognitive Status**				$\chi^2 = 0.21$	0.650
Without objective cognitive impairment	138 (68.32%)	67 (66.34%)	71 (70.30%)		
Cognitively impaired	64 (31.68%)	34 (33.66%)	30 (29.70%)		

**Depressionbefore**	2.00 (1.00, 4.00)	2.00 (1.00, 4.00)	2.00 (1.00, 4.00)	Z = 4928	0.675
**Anxietybefore**	0.00 (0.00, 4.00)	0.00 (0.00, 3.00)	0.00 (0.00, 4.00)	Z = 5068.5	0.933
**AD8**	2.00 (2.00, 3.00)	2.00 (2.00, 3.00)	2.00 (1.00, 4.00)	Z = 4655.5	0.267
**CSID**	8.00 (7.00, 8.00)	8.00 (7.00, 8.00)	8.00 (7.00, 9.00)	Z = 4950	0.709

Notes: χ^2: Chi-square test; Z :Wilcoxon rank sum test

AD8 : Ascertain Dementia 8-item Questionnaire; CSI-D: Community Screening Instrument for Dementia

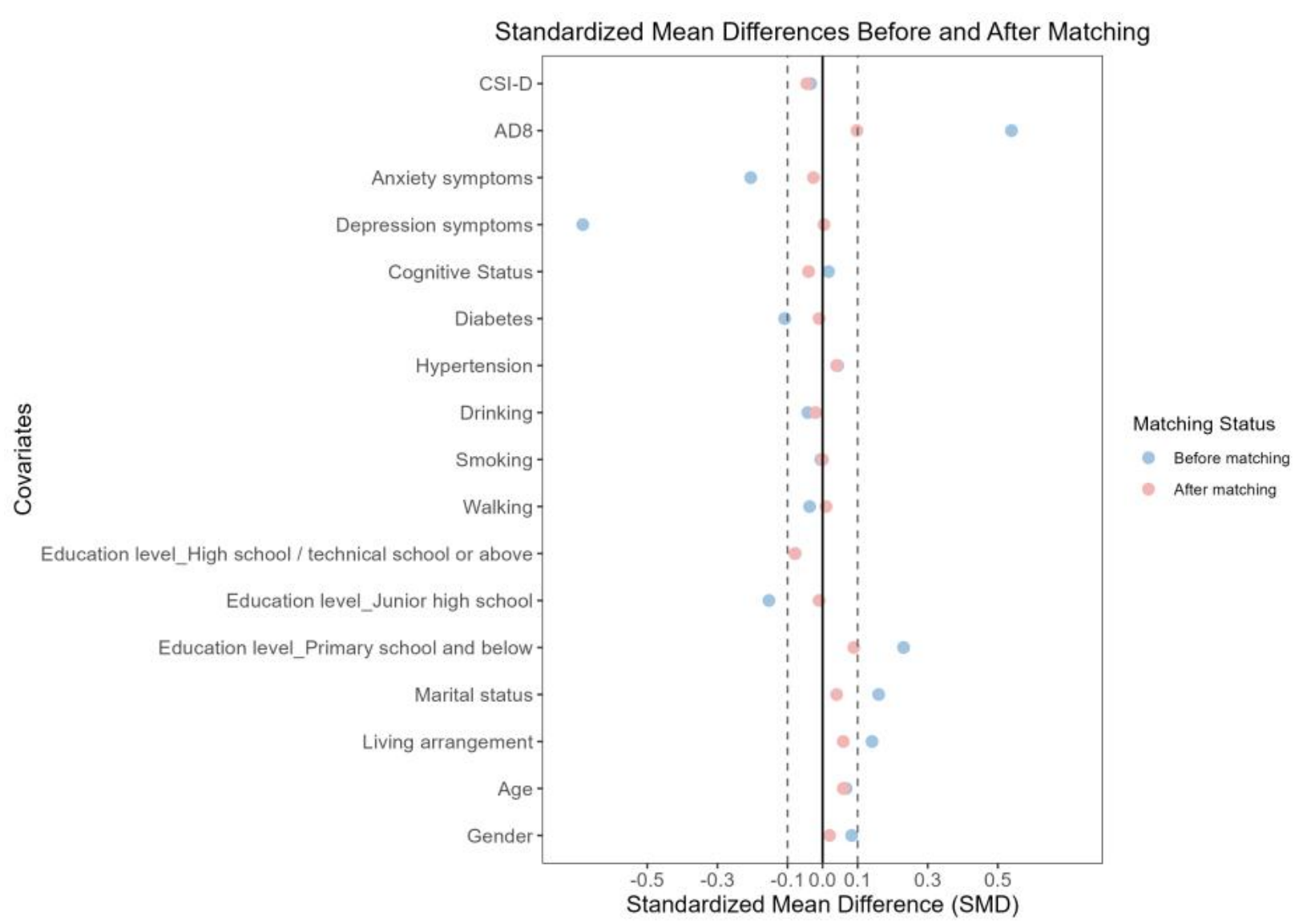

Figure S1. Standardized mean differences of covariates before and after propensity score matching
